# Sensing Cd(II) Using a Disposable Optical Sensor Based on a Schiff Base Immobilisation on a Polymer-Inclusion Membrane. Applications in Water and Art Paint Samples

**DOI:** 10.3390/polym13244414

**Published:** 2021-12-16

**Authors:** Lorena Sánchez-Ponce, María Dolores Galindo-Riaño, María José Casanueva-Marenco, María Dolores Granado-Castro, Margarita Díaz-de-Alba

**Affiliations:** Department of Analytical Chemistry, Institute of Biomolecules (INBIO), Faculty of Sciences, CEI-MAR, Campus Río San Pedro, University of Cádiz, ES-11510 Puerto Real, Spain; lorena.sanchezponce@alum.uca.es (L.S.-P.); dolores.galindo@uca.es (M.D.G.-R.); mariajose.casanueva@uca.es (M.J.C.-M.); margarita.diaz@uca.es (M.D.-d.-A.)

**Keywords:** polymer-inclusion membrane, Cd(II) ions determination, optical sensor, PVC matrix, 2-acetylpyridine benzoylhydrazone

## Abstract

A disposable colour-changeable optical sensor based on an interesting polymer inclusion-membrane (PIM) was designed to determine Cd(II) ions in aqueous medium. The Schiff base 2-acetylpyridine benzoylhydrazone (2-APBH) immobilised on the polymer membrane was used as a sensing molecule. The amounts of the PIM components were optimised by a 3^2^ fractional factorial design with two central points and two blocks. The best optical sensor composition consisted of 2.5 g of poly(vinylchloride) (PVC) as a base polymer, 3 mL of tributyl phosphate (TBP) as a plasticiser, and 0.02 g of 2-APBH as a reagent. The sensor showed a good linear response in the range from 0.02 mg L^−1^ (limit of detection) to 1 mg L^−1^ of Cd(II) under the following experimental conditions: pH 9.5 (adjusted using ammonium chloride buffer solution at 0.337 mol L^−1^), 60 min of exposure time plus 2 min of sonication (pulses at 2 s intervals), and 10 min of short-term stability. The relative standard deviation of the method was determined to be 4.04% for 0.4 mg L^−1^ of Cd(II). The optical sensor was successfully applied to the determination of Cd(II) in natural-water and art-paint samples.

## 1. Introduction

A chemical sensor is a device that responds to a particular analyte in a selective way through a chemical reaction and can be used for its qualitative or quantitative determination. According to the traducer type, they can be categorised into: electrochemical, optical, mass sensitive, and heat sensitive [1]. Optical chemical sensors (often referred to as optodes) are a low-cost alternative to conventional methods, with high sensitivity and selectivity, fast response, and simple instrumentation. The response can be based on the absorption, emission, reflectance, photoluminescence, or chemiluminescence associated with the chemical interaction [1,2,3,4,5]. Membranes play a very important role in chemical sensing. The application of polymer-inclusion membranes (PIMs) as sensing components in ion-selective electrodes (IEs) and optodes [6,7,8] has generated great interest. PIMs have also been used for separation [9,10], preconcentration [11], and passive sampling [12,13], resulting in a functional, cheaper, and more environmentally friendly alternative methodology [14].

PIMs essentially consist of three components: a base polymer, a reagent (to facilitate the binding with the species of interest), and a plasticiser. They are generally prepared by the solution casting method, where these components are dissolved in an organic volatile solvent, such as tetrahydrofuran (THF), 2-metiltetrahydrofuran (MeTHF), or dichloromethane (DMC). The solvent is finally evaporated, and a thin polymeric film is formed [15]. The base polymer gives stability to the membrane, where poly(vinyl chloride) (PVC) and cellulose triacetate (CTA) are some of the most used. The reagent immobilised into the PIM allows the membrane–analyte interaction and the plasticiser confers flexibility to the membrane [6,16,17]. Other possible components are additives, such as anion or cation exchangers (trioctylmethylammonium chloride (Aliquat 336), dinonylnaphthalene sulphonic acid (DNNS), etc.), which may improve the selective extraction of the analyte [18,19,20]. Depending on the base polymer, reagent, plasticiser, modifier, and additive used in the synthesis, PIMs can show different physicochemical properties.

PIMs have been used as optical chemical sensors for the selective separation and determination of metal ions by spectrophotometry (for example, Zn(II) determination in water, food supplements, and foot healthcare products [21]; Al(III) in aqueous samples [16]; or Cd(II), Zn(II), and Cu(II) in real water samples [22]), spectrofluorometry (such as Fe(III) in real samples [23] and Al(III) in natural water [24,25]), or colour-intensity measurements (such as Cu(II) [26] and Ag(I) and Hg(II) [27] in water samples), among others. In this sense, the use of Schiff bases in PIMs design can be very interesting since they are very effective chelating reagents used for the analysis, removal, and recovery of metal ions. Among them, hydrazones are characterised by the presence of the group =C=N–N=, offering good optical properties after the complexation of metal ions such as Ni, Co, Zn, Cd, or Cu [28].

Some heavy metals may be very toxic at low concentrations, such as arsenic, cadmium, chromium, mercury, or lead, which are known as non-essential trace elements [29]. Cadmium is one of the heavy metals with major environmental risk [30]; moreover, it has been recognised as a human carcinogen by the World Health Organization. Usually, cadmium is a naturally occurring element present in all soils from both geogenic (as natural component of rocks) and anthropogenic sources (0.16 mg kg^−1^ in the Earth’s crust [31]). Many anthropogenic processes, such as pigment, ceramic and battery production, and agriculture, among others, cause the increase in cadmium concentration in air, soil, and water [32]. This increase and the high persistence of its compounds in the environment produce a greater risk for living organisms exposed to cadmium [33,34]. In 2014, the European Union stated the tolerable cadmium intake per week in 2.5 μg kg^−1^ of body weight [35]. Cadmium intoxication may lead to renal tubular injury [36], hepatic, skeletal and cardiovascular damage, and even cancer [37]. Humans can be daily exposed to Cd via air, drinking water, or food. Different studies have described this exposure due to the intake of some infant formulas and baby foods [38], cereals [39], small bivalves and crustaceans [40], cigarettes smoke [41], handling of batteries [42], or the use of paints and articles coloured with pigments such as toys, ceramics, plastics, or decorated glassware [43].

There are some studies regarding the use of PIMs for selective extraction of cadmium ions [44,45,46], but very few studies are focused on its application as an optical sensor. Sanchez-Pedreño et al. [47] studied three kinetic methods using 1-(2-pyridylazo)-2-naphtol (PAN)-PVC-NPOE as a reagent for a Cd-based PIM sensor applied to Wood’s alloy and water samples. These measurements were carried out by means of diffuse-reflectance spectroscopy. The same reagent was used for a PIM-based optical sensor for Cd detection in natural waters [48] and for the quantitatively determination of Cd(II), Zn(II), and Cu(II) in wastewater samples from a nonferrous metal smelter by UV–Vis spectrophotometry [22].

**Figure 1:**
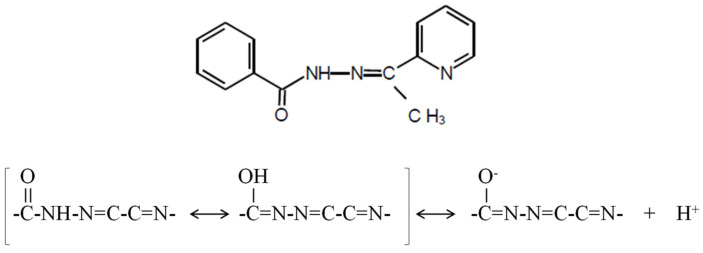
The chemosensor 2-acetylpyridine benzoylhydrazone (2-APBH) and its tautomeric structures [49].

The aim of this study was the design of a polymer-inclusion membrane (PIM) as an optical sensor to determine Cd(II) ions using the Schiff base 2-acetylpyridine benzoylhydrazone as chemosensor (2-APBH; molecular formula: C_14_H_13_N_3_O; molecular weight: 239.3; mp: 154–156 °C). This reagent forms chromogenic complexes with different transition metal ions such as Cd(II), Ni(II), Cu(II), Pb(II, Ti(IV), Fe(III), Fe(II), etc. [49]. It is a biologically active aroylhydrazone with antibacterial and antifungal activities that has been used as antitubercular drug [50]. This ligand shows a maximum absorption at 300 nm in water solution with a bathochromic shift at alkaline conditions. The pK values in aqueous medium are found to be 3.6 ± 0.3 and 11.1 ± 0.1 at 0.1 mol L^−1^ ionic strength. The protonation of the pyridine N atom may be associated with the first pK, and the deprotonation of –NHCO– group corresponds with the second pK. This heterocyclic ligand exhibits two tautomeric structures (carbonyl–enol group) and can form coordination complexes through the oxygen atom of the enol form, the azomethine nitrogen, and/or the acetylpyridine ring nitrogen (Figure 1). Thus, it can act as a bidentate or tridentate ligand producing a five-membered ring [49].

The complexation reaction is highly influenced by the pH of the solution, and it is favoured at neutral or alkaline media in which the –NHCO– group can be deprotonated. The hydrolysis of the ligand can take place at low pH values, but it can be avoided using acetate or ammonia buffer solutions. The reagent 2-APBH and Cd(II) ion forms a stable yellow complex showing the maximum absorbance at 345 nm in neutral solution with a metal:ligand ratio stoichiometry of 1:2 [51]. Thus, this ligand presents interesting properties to be used as an optical chemosensor for Cd ions.

## 2. Materials and Methods

### 2.1. Reagents and Solutions

All reagents and solvents were of analytical-reagent or Suprapur grade, and all the solutions were prepared using ultra-high-quality water. Aqueous solutions of Cd(II) were prepared using a cadmium ICP standard solution of 1000 mg L^−1^ (Merk, Darmstadt, Germany) in 0.05 mol L^−1^ HNO_3_ (Merk, Darmstadt, Germany). Ammonium-chloride buffer solutions (3 mol L^−1^, pH 8–10) were prepared using ammonia solution (25%) and hydrochloric acid (37%) (Merk, Darmstadt, Germany). In order to increase the ionic strength of metal solutions, NaNO_3_ (analytical grade, D’Hemio Laboratorios, Carabanchel, Madrid Spain) and NaClO_4_ (prepared from HClO_4_ and NaOH (analytical grade, Panreac, Castellar del Vallès, Barcelona, Spain) were used. To digest the acrylic paint, HNO_3_ (65%), H_2_O_2_ (30%), and HClO_4_ (70%) (Suprapur grade, Merk, Darmstadt, Germany) were used.

For the synthesis of the PIM, poly(vinyl chloride) (PVC) (Sigma-Aldrich, St. Louis, MO, USA) as a base polymer, tributyl phosphate (TBP) (Sigma-Aldrich, St. Louis, MO, USA) as a plasticiser, tetrahydrofuran (THF) (Panreac, Castellar del Vallès, Barcelona, Spain) as a volatile solvent, and 2-acetylpyridine benzoylhydrazone (2-APBH) as the chromogenic reagent were used. The Schiff base (2-APBH) was synthesised by reaction of 2-acetylpyridine (Sigma-Aldrich, St. Louis, MO, USA) and benzoylhydrazine (Sigma-Aldrich, St. Louis, MO, USA) [50].

Interferences were studied by using stock aqueous solutions of Ag(I), Al(III), As(V), Bi(III), Cr (III), Cu(II), Fe(II), Fe(III), K(I), Ni(II), Pb(II), Se(II), Ti(IV), Tl(I), V(IV), and V(V). These solutions were prepared from an ICP standard solution of 1000 mg L^−1^ in 0.05 mg L^−1^ HNO_3_ (Certipur, Merk, Darmstadt, Germany). Fe(II) was prepared from Fe(III) solution in the presence of ascorbic acid (4.75 mg L^−1^ ascorbic acid: 1 mg L^−1^ Fe(III) ratio).

### 2.2. Instrumentation

Ultrapurified water was obtained by reverse osmosis with an Autwomatic (Water type II) system followed by ion exchange with an 18.2 MΩ cm deionised Ultramatic Plus system (Wasserlab, Barbatáin, Navarra, Spain). Reagents and aqueous solutions were prepared under an 870-FL vertical laminar flow cabinet (Cruma, Saint Boi de Llobregat, Barcelona, Spain). The pH measurements were conducted using a Basic 20 pH-meter with a 50_10T glass-Ag/AgCl electrode (Crison, Barcelona, Spain). The samples were shaken by means of an HS 501 D shaker platform (Ika, Labortechnik, Staufen, Germany). The polymer-inclusion membranes were synthesised inside a fume hood (Flowtronic, Romero S.A., Torrejón de Ardoz, Madrid, Spain), and an ultrasonic bath (Ultrasons, 9L, J.P. Selecta, Lardero, La Rioja, Spain) was used in order to homogenise and dissolve the PIM components. A Q700 high-energy ultrasound generator (Qsonica Sonicators, Newtown, CT, USA) was used for the sonication of the metal solutions in contact with the PIM. A handmade polyester film support (thickness: 25 μm, Mylar^®^, Dupont, Hopewell, VA, USA) was used for the UV–Vis measurements of the PIM due to its special optical characteristics. The absorbance values were measured with a high-quality V-650 double beam UV–Vis spectrophotometer (Jasco, Hachioji, Tokio, Japan) controlled by Spectra ManagerTM software (Jasco, Hachioji, Tokio, Japan). Real samples were digested in vessels (Teflon autoclaves) under a controlled temperature by an ETHOS 1 Advanced Microwave Digestion System (Milestone, Sorisole, Bergamo, Italy). In order to prove the accuracy and reliability of the method, real samples were analysed by both flame atomic absorption spectroscopy using a Thermo Scientific ICE 3000 Series AA Spectrometer (Thermo Fisher Scientific, Winsford, UK) and the proposed sensor.

### 2.3. Membrane Preparation

The potential use of the reagent 2-APBH as a sensing molecule immobilised in a polymeric membrane of PVC (base polymer) and TBP (plasticizer) was proposed in a previous work. These components provide a homogeneous, flexible, mechanically strong, and optically transparent membrane [21]. In this study, the amounts of PIM components for Cd determination were optimised by applying a 3^(k−1)^ factorial design with two repetitions of the central point in two blocks. This chemometric tool allows evaluating the effects of the experimental factors and their interactions on the response of the optical sensor. For that, the amounts of components for each experiment of the design were dissolved in 19.1 mL of THF (volatile solvent) by means of an ultrasonic bath until a homogeneous solution was obtained. The mixture was then deposited on a glass Petri dish of 11.7 cm diameter, which was covered with a piece of filter paper to allow THF to evaporate into a dark fume hood for a curing time of 24 h at a controlled room temperature of 20 °C. After that, the membrane obtained was cut into rectangular portions (2.37 cm × 0.98 cm size) and assembled onto a piece of polyester film (4.75 cm × 0.98 cm size). This ensemble was used as an optical sensor (optode) and was stored into a desiccator in the absence of light until its use. The absorbance data from the Cd sensing experiments were obtained using Spectra Manager^TM^ software (Jasco, Hachioji, Tokio, Japan) and then exported to Microsoft Excel 2016 (Microsoft Corporation, Redmond, WA, USA) for data processing. The results of the experimental design were analysed using the software Statgraphics Centurion XVII (Statpoint Technologies, Inc., The Plains, VA, USA).

### 2.4. Analytical Procedure for Optical Sensing of Cd(II) Ions

The batch experiments were based on letting the optical sensor equilibrates with the Cd(II) sample solution and the subsequent measurement of the absorbance of the complex formed between Cd(II) and the Schiff base (2-APBH) by UV–Vis spectrophotometry. For each experiment, the optical sensor was immersed into 20 mL of Cd(II) solution (or blank solution) at pH 9 (buffered by 0.225 mol L^−1^ of ammonium chloride/ammonia solution) using 100 mL polypropylene containers. The container was shaken at 300 rpm for 60 min at 20 °C. Afterwards, the sensor was taken out of the container, rinsed with ultrapurified water, dried with absorbent paper, and stored for 10 min into a desiccator in the absence of light. The pH of the sample solution was controlled by measuring it before and after the exposure to the sensor. Finally, the optical sensor was perpendicularly placed into a quartz cuvette, and the absorbance was measured by a UV–Vis spectrophotometer at 379 nm (wavelength of maximum absorption of the Cd(II)-2-APBH complex). A piece of the polyester film was used as a reference measurement. The difference between the absorbance of the sensor after and before the experiment was defined as the sensor response. All experiments were carried out in duplicate.

### 2.5. Real Samples Preparation

In order to ensure the accuracy and reliability of the proposed method for the determination of Cd(II) ions, real samples were analysed by using the designed optical sensor under the optimal conditions. The results were compared to the ones obtained by AAS. The analytical procedures to prepare the real samples were as follows:

(a) Spiked groundwater
  Groundwater-certified reference material (BCR^®^-610) (Joint Research Centre (JRC), Institute for Reference Materials and Measurements (IRMM), with a certified value for Cd of 2.94 ± 0.08 μg kg^−1^) was directly used for assessing the method performance.
(b) Art-paint samples
  Two school acrylic art-paint samples were analysed: “cadmium yellow orange” (P020 colour index) and “cadmium red deep” (PR108 colour index). For that, 0.1 g of each paint were digested using 1 mL of HNO_3_ (65%), 1 mL of H_2_O_2_ (30%), and 1 mL of HClO_4_ (70%) in a Teflon vessel (waiting 24 h between the addition of each reagent (HNO_3_, H_2_O_2_, and HClO_4_); vessels were kept closed during that time). Two steps were followed for the digestion of the sample in a microwave oven at these conditions: 800 W, 120 °C for 10 min and 800 W, 170 °C for 30 min. The solution was made up to 25 mL with ultrapurified water. The final solutions for both art paints were diluted to obtain four different samples called acrylic paint 1, 2, 3, and 4, where 1 and 2 were two different dilutions from “yellow cadmium orange” paint and 3 and 4 were two different dilutions from “dark red cadmium” paint. These four samples were analysed following the new methodology proposed in this work for the determination of Cd(II) ions.

## 3. Results

### 3.1. Optimisation of the Optical Sensor Composition and Curing Time

**Figure 2:**
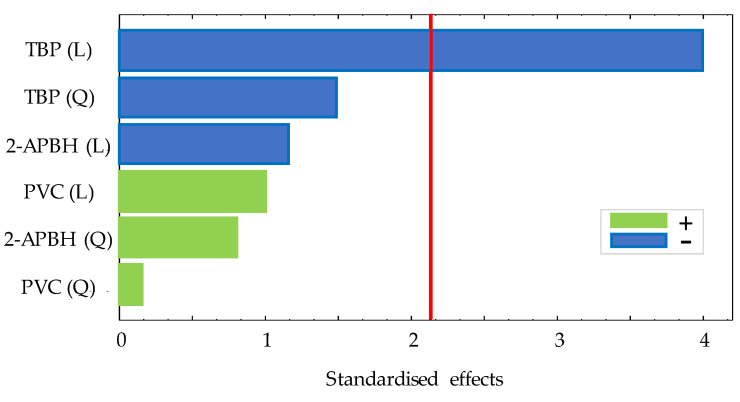
Pareto chart for 3^(3−1)^ experimental design for optical sensor response (red line: significance level of 5%).

In order to study the optimal composition of the optical sensor, a 3^(k−k)^ fractional factorial design with two central points and two blocks was performed studying three variables (amount of PVC (g), volume of TBP (mL), and amount of 2-APBH (g)) by following the procedure described in Section 2.3. The exponent k indicates the number of variables, and the base indicates the levels of each variable. All variables presented three levels: an upper level (+1), a central level (0), and a lower level (−1). The corresponding values of each level for the different variables are shown in Table 1. A total of 22 random runs were performed: each membrane composition was prepared twice in different days, and, in turn, the exposure experiment for each membrane was carried out in duplicate. All the experiments were carried out by exposing the sensor to 1 mg L^−1^ of Cd(II) solutions at pH 9 (buffered by 0.225 mg L^−1^ ammonium chloride/ammonia solutions). The difference between the absorbance measurement of the sensor before and after the exposure time (30 min) to the solution is defined as the response of the sensor. Table 2 shows the matrix of each experiment, the response of the sensor exposed to Cd(II), and the standard deviation of these measurements. The significance of the effects of the three variables was studied for a 95% confidence interval. The analysis of the experimental design showed non-significant interactions between the effects of the sensor exposed to Cd(II) on the response. The Pareto chart for the lineal (L) and quadratic (Q) main effects is shown in Figure 2.

**Figure 3:**
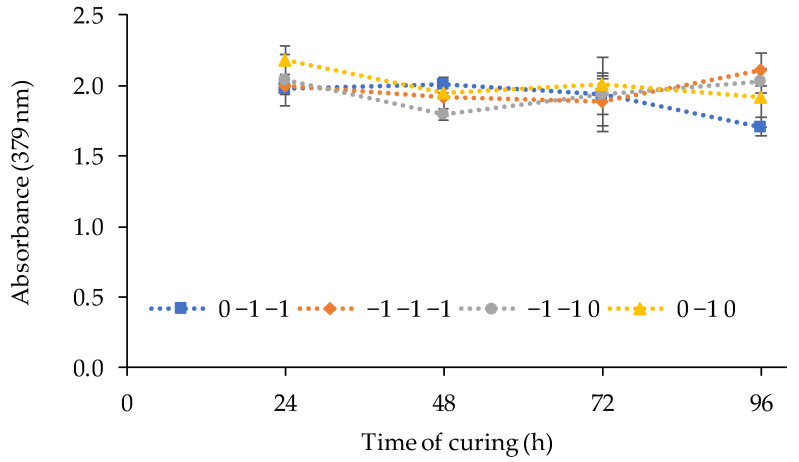
Effect of the curing time on the sensor response (exposed to 1 mg L^−1^ of Cd(II) solutions at pH 9) for different combinations of PVC and 2-APBH levels.

According to it, the volume of TBP showed a significant negative linear effect on the response, being the optimum factor level as (−1). The amount of PVC and 2-APBH showed a non-significant effect. The best PIM composition to be used for Cd(II) sensing combines the highest response of the sensor exposed to cadmium, the lower reagent consumption, and the lower standard deviation between replicates, although the two last effects were not significant. For that purpose, the synthesis and the curing time of the sensor from different runs ((PVC, TBP, 2-APBH): −1, −1, −1; 0, −1, −1; −1, −1, 0; and 0, −1, 0) with the reagent consumption as low as possible (level (−1) and level (0)) was evaluated. Figure 3 shows the response of the sensor for different combinations of PVC and 2-APBH levels after exposure to 1 mg L^−1^ of Cd(II) solution at pH 9 for 24, 48, 72, and 96 h of curing time.

The results showed in most cases a decrease in the sensor signal after the first 48 h of curing. On the other hand, the best stability of the signal, and therefore the lower deviation between replicates for the first 48 h, corresponded to the synthesis of the PIM with the central level of PVC (0) and the lower level of 2-APBH (−1). Thus, the optimal PIM composition to obtain the best response of the sensor was established in: 2.5 g of PVC, 3 mL of TBP, and 0.02 g of 2-APBH. In addition, the curing of the optical sensor can be completed within the first 24–48 h after the synthesis because, for 72 h, a decrease was observed.

### 3.2. Lifetime of the Optical Sensor

The lifetime of the Cd(II) optical sensor is a fundamental aspect for the experimentation because it provides information about how many days the sensor can be used after the synthesis. A PIM (2.5 g PVC; 3 mL TBP; 0.02 g 2-APBH) was synthesised following the procedure described in Section 2.3 in order to determinate its lifetime. The absorbance of the optical sensor due to the Schiff base (maximum absorption at 324 nm) was measured at 6, 12, 24, 30, 48, 96, and 168 h after synthesis. The results for two replicates showed constant values of the absorbance up to 168 h (see Appendix A: Table A1). A *t*-test at a 95% confidence interval was performed with the data obtained at 6 and 168 h, indicating there were not significant differences between them. Therefore, this study proved that the sensor could be used up to seven days after synthesis.

### 3.3. Effect of pH, Buffer Concentration, and Ionic Strength on the Response of the Sensor

**Figure 4:**
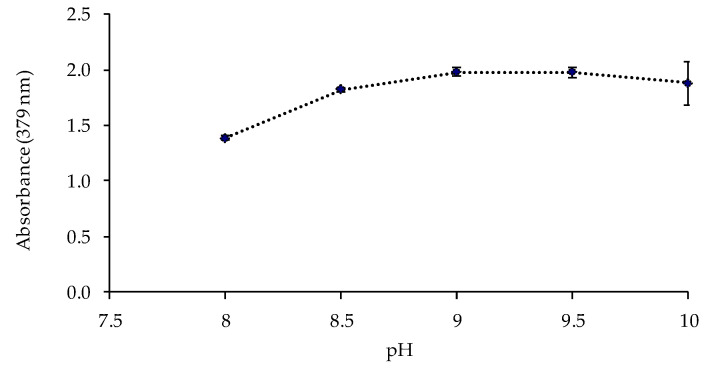
Effect of the pH value of the ammonium chloride/ammonia buffer solution on the sensor response (1 mg L^−1^ Cd(II); 0.225 mol L^−1^ buffer; n = 2).

**Figure 5:**
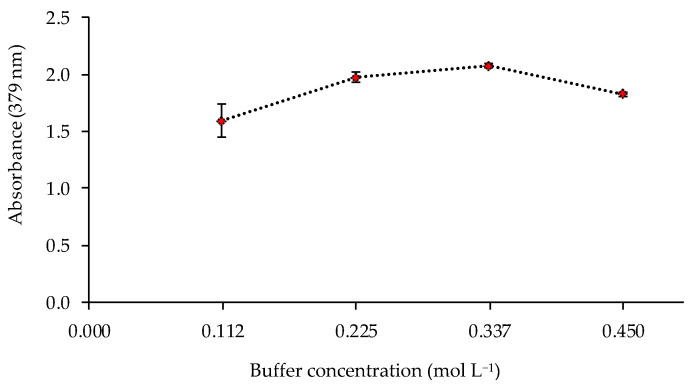
Effect of the buffer concentration on the sensor response (1 mg L^−1^ Cd(II); pH 9.5; n = 2).

The pH value and the buffer concentration in the metal solution could affect the response of the sensor. Thus, the sensor was exposed to solutions of 1 mg L^−1^ of Cd(II) at different pH values (8, 8.5, 9, 9.5, and 10) controlled by 0.225 mol L^−1^ ammonia buffer solutions. The best responses of the sensor were obtained at pH 9–9.5 (Figure 4). The influence of the buffer concentration was also studied at pH 9.5, obtaining a higher absorbance value for a buffer concentration of 0.337 mol L^−1^ (Figure 5).

**Figure 6:**
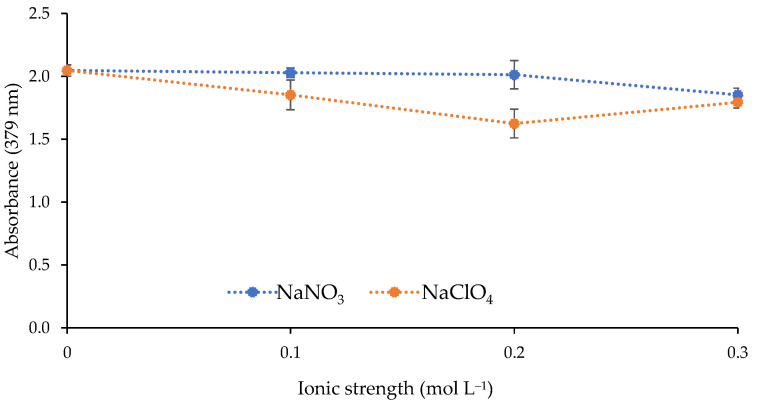
Effect of the ionic strength on the sensor response (1 mg L^−1^ Cd(II); pH 9.5; 0.337 mol L^−1^ buffer; n = 2).

The effect of the ionic strength of the metal solution on the response of the sensor was investigated. Two different salts (NaNO_3_ and NaClO_4_) were used to increase the ionic strength of the solution by adding the following concentrations: 0, 0.1, 0.2, and 0.3 mol L^−1^. As shown in Figure 6, the increase in ionic strength did not improve the sensor response.

### 3.4. Response Time of the Optical Sensor

**Figure 7:**
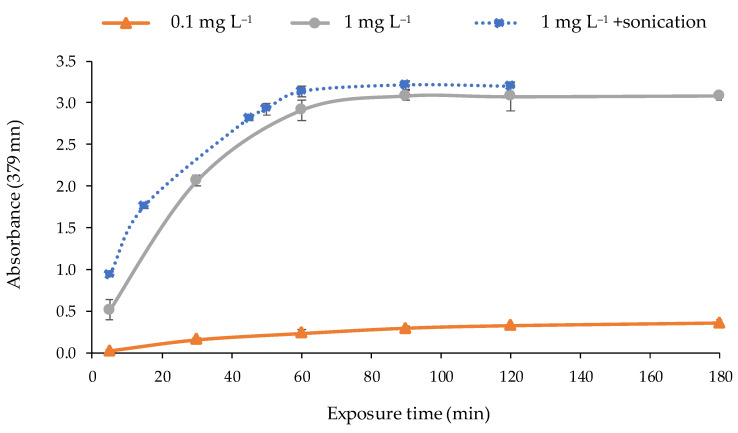
Response time of the optical sensor for different concentrations of Cd(II) (pH 9.5; 0.337 mol L^−1^ buffer; n = 2) exposed to: shaking (―); shaking + sonication (2 min) (·····).

The response time of the optical sensor was studied to determinate the adequate time of exposure to the metal solution required to obtain a maximum and stable signal. For that purpose, the optical sensor was exposed to solutions of 1 and 0.1 mg L^−1^ of Cd(II) under the optimal conditions during different times (5, 30, 60, 90, 120, and 180 min) by shaking at 300 rpm. According to the results (Figure 7), the exposure time to obtain the maximum response was 90 min. After that, the response (absorbance value) remained constant. A sonication process of the optical sensor in the solution with a power of 21 W on a discontinuous mode (2 min of sonication pulses at 2 s intervals) was applied after shaking to promote the complexation of the metal by the PIM. As can be seen in Figure 7, a rise in the response time was observed since the sonication time reduced the metal exposure in 30 min.

No longer sonication times were studied because the temperature of the solution was increased in excess, and the reagent is not thermally stable. The hydrolysis of 2-APBH occurs when temperature exceeds 35–40 °C [49,51]. Therefore, the selected exposure time to the metal solution was 60 min followed by sonication for 2 min on a discontinuous mode.

### 3.5. Short-Term Stability

**Figure A1:**
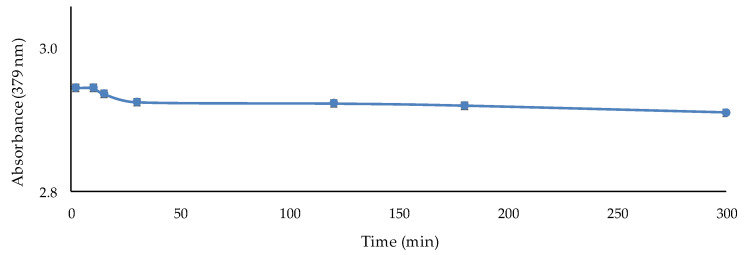
Study of the short-term stability of the optical sensor exposed to 1 mg L^−1^ Cd(II).

Short-term stability is the period of time that can elapse between the end of the exposure of the sensor to the metal solution and the absorbance measurement in the spectrophotometer without changes in the sensor response. This study was performed by exposing the sensor to 1 mg L^−1^ Cd(II) at optimal conditions (PIM composition: 2.5 g PVC, 3 mL TBP, and 0.02 g 2-APBH; conditions of the metal solution: pH 9.5 and 0.337 mol L^−1^ buffer solution concentration; 60 min of exposure time followed by 2 min of sonication). The absorbance was measured during 5 h after sonication. The results (Appendix A: Figure A1) showed a slight decrease over time after the first 15 min and a constant signal in the range of 30–120 min with a slight decrease again. Therefore, a 10 min short-term stability was selected as the waiting time before spectrophotometric measurement was performed, but any time within the interval from 30 to 120 min would also be applicable.

### 3.6. Analytical Performance of the Method

**Figure 8:**
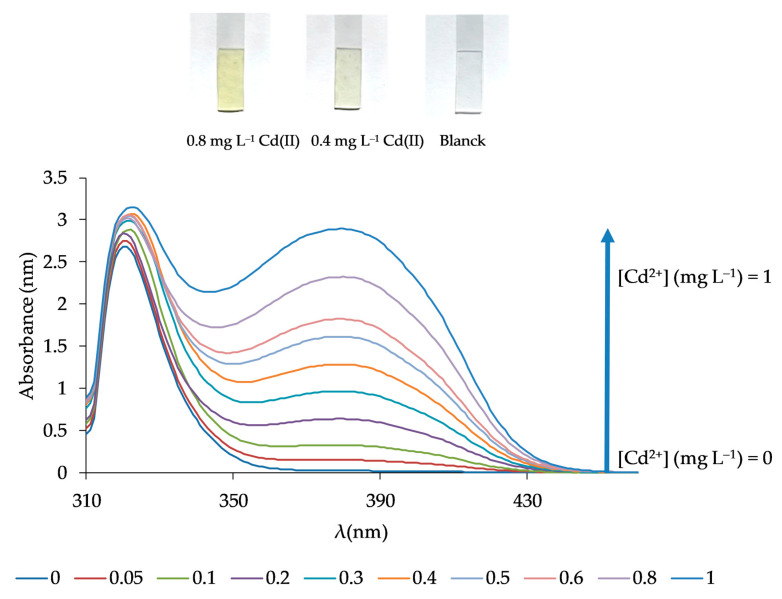
Response spectra of the optical sensor for different Cd(II) concentrations corresponding to the calibration curve.

The analytical features of the method such as the limit of quantification (LQ) and detection (LD), the dynamic linear range, the precision, and the selectivity were determined. For that, the response of the sensor was obtained following the procedure previously described in Section 2.4 under optimal conditions. The LD was evaluated using 3σ/m (n = 10), where σ is the standard deviation of the blank signal, and m is the slope of the linear calibration plot. The calculated LD was 0.021 mg L^−1^. The LQ (evaluated as 10σ/m (n = 10)) was 0.069 mg L^−1^. The relationship between the signal and the Cd(II) concentration was found to be linear over the concentration range from 0.05 to 1 mg L^−1^ with an equation of (Equation (1)):

Abs (379 nm) = (2.884 ± 0.012) [Cd(II)](mg L^−1^) + (0.046 ± 0.006) (1)

obtaining a correlation coefficient of R^2^ = 0.9966 (n = 10) and a standard error of estimate of 0.059 (Figure 8). The precision of the method, calculated from six replicate experiments using 0.4 mg L^−1^ Cd(II) solutions at a confidence interval of 95%, was 3.38%, while the relative standard deviation was found to be 4.04%.

**Figure 9:**
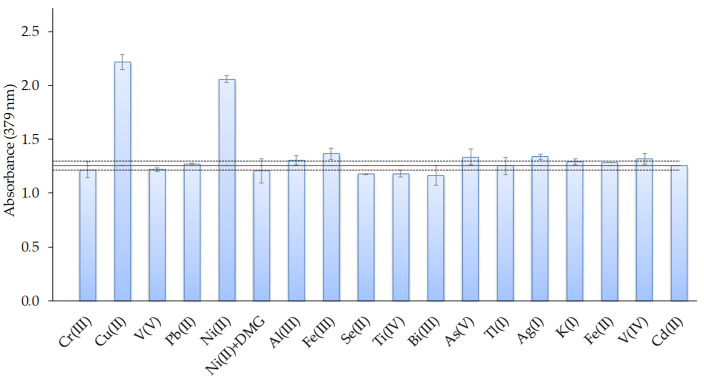
Effect of the presence of different metal ions on the response of the optical sensor for a 1:1 Cd(II): interfering metal ratio (metal concentrations: 0.4 mg L^−1^; n = 2).

The selectivity of the sensor was investigated as a function of the degree of interferences found in the presence of different ions such as Ag(I), Al(III), As(V), Bi(III), Cr (III), Cu(II), Fe(II), Fe(III), K(I), Ni(II), Pb(II), Se(II), Ti(IV), Tl(I), V(IV), and V(V). For that, the effects of these ions on the response of the sensor were evaluated at a 1:1 Cd(II): interfering ion ratio, using metal ion concentrations of 0.4 mg L^−1^. The tolerance limit was defined as the concentration of the added ion causing a relative error less than ±5% in the optical-sensor response. The results are shown in Figure 9 and indicated that most of the studied ions produced low interference or did not affect the Cd response, except for Cu(II) and Ni(II) ions. The pre-treatment of the metal solution with dimethylglyoxime (DMG: 0.068 mmol L^−1^) addition and filtration before Cd(II) analysis avoided the Ni(II) interference (Figure 9).

### 3.7. Analytical Applications

In order to evaluate the accuracy of the method, spiked groundwater samples with different amounts of Cd(II) and acrylic art paint samples were analysed by using the proposed optical sensor. After that, the results were compared to those values obtained by AAS by means of a Student’s t-test at a 95% confidence interval. As shown in Table 3, there were no significant differences between both methods (optical sensor and AAS technique) in the quantitative determination of Cd(II). Therefore, the proposed optical sensor can provide reliable results and be used for the successful determination of Cd(II) ions in real samples such as waters and paints.

## 4. Conclusions

A new optical sensor for Cd(II) determination was successfully developed and applied. The proposed sensor is based on the immobilisation of a Schiff base (2-APBH, a colorimetric chelating reagent) in a polymer-inclusion membrane (PIM). The sensor has been optimised and characterised by using spectrophotometric measurements. It offers good mechanical and optical properties, such a good reproducibility, precision, and selectivity with an easy and low-cost operation. The optical sensor showed good potential applicability and provided reliable results when analysing spiked groundwater and art-paint samples with different concentrations of Cd(II).

## Figures and Tables

**Table 1:** Values of each level for the three variables studied.

Variable	Level		
	Lower Level (−1)	Central Level (0)	Upper Level (+1)
PVC (g)	2	2.5	3
TBP (mL)	3	4	5
2-APBH (g)	0.02	0.03	0.04

**Table 2:** Matrix of the 3^3−1^ experimental design (22 runs; n = 2) and response of the optical sensor exposed to 1 mg L^−1^ of Cd(II) at pH 9 (n = 2).

Replicates of Composition	Experiment	PVC (g)	TBP (mL)	2-APBH (g)	Abs (379 nm)	
					Mean	SD
1	1	−1	−1	−1	2.038	0.119
1	2	−1	0	1	1.682	0.534
1	3	−1	1	0	1.536	0.005
1	4	0	−1	1	1.880	0.044
1	5	0	0	0	1.695	0.059
1	6	0	1	−1	1.691	0.011
1	7	1	−1	0	2.117	0.008
1	8	1	0	−1	1.712	0.009
1	9	1	1	1	1.601	0.006
1	10	0	0	0	1.826	0.421
1	11	0	0	0	1.488	0.016
2	12	−1	−1	−1	1.892	0.142
2	13	−1	0	1	1.321	0.149
2	14	−1	1	0	1.694	0.128
2	15	0	−1	1	1.962	0.232
2	16	0	0	0	2.036	0.159
2	17	0	1	−1	1.477	0.178
2	18	1	−1	0	1.846	0.172
2	19	1	0	−1	1.832	0.510
2	20	1	1	1	1.591	0.200
2	21	0	0	0	1.609	0.304
2	22	0	0	0	1.697	0.050

**Table 3:** Analysis of Cd(II) in real samples by the proposed optical sensor and AAS technique.

Sample	(Cd(II)) ± SD (mg L^−1^)		t_calc_	t_tab_ (95%)
	Optical Sensor	AAS		
Spiked groundwater with 0.3 mg L^−1^	0.309 ± 0.011	0.300 ± 0.002	1.440	3.182
Spiked groundwater with 0.8 mg L^−1^	0.802 ± 0.001	0.806 ± 0.002	2.425	3.182
Acrylic paint 1	0.544 ± 0.003	0.538 ± 0.009	0.878	3.182
Acrylic paint 2	0.680 ± 0.013	0.664 ± 0.002	2.237	3.182
Acrylic paint 3	0.478 ± 0.001	0.485 ± 0.004	1.935	3.182
Acrylic paint 4	0.732 ± 0.047	0.743 ± 0.001	0.436	3.182

## Data Availability

All data from this research are included in the article.

## Appendix A

**Table A1:** Lifetime of the optical sensor based on the absorbance measurements for several days after the synthesis of the PIM.

Time (h)	Replicate	Absorbance (324 nm)	Absorbance Average
6	1	2.9883	2.9789
	2	2.9694	
12	1	2.9872	2.9791
	2	2.9709	
24	1	2.8851	2.9310
	2	2.9769	
30	1	2.9916	2.9846
	2	2.9775	
48	1	3.0012	2.9579
	2	2.9146	
96	1	2.9864	2.9937
	2	3.0009	
168	1	3.0087	3.0105
	2	3.0123	
